# Effects of amiloride on acetylcholine‐dependent arterial vasodilation evolve over time in mice on a high salt diet

**DOI:** 10.14814/phy2.15255

**Published:** 2022-04-06

**Authors:** Stephanie M. Mutchler, Thomas R. Kleyman

**Affiliations:** ^1^ Department of Medicine University of Pittsburgh Pittsburgh Pennsylvania USA; ^2^ Departments of Cell Biology and of Pharmacology and Chemical Biology University of Pittsburgh Pittsburgh Pennsylvania USA

**Keywords:** amiloride, ENaC, endothelium, salt sensitivity

## Abstract

The maintenance of endothelial health is required for normal vascular function and blood pressure regulation. The epithelial Na^+^ channel (ENaC) in endothelial cells has emerged as a new molecular player in the regulation of endothelial nitric oxide production and vascular stiffness. While ENaC expression in the kidney is negatively regulated by high [Na^+^], ENaC expression in isolated endothelial cells has been shown to increase in response to a high extracellular [Na^+^]. In culture, this increased expression leads to cellular stiffening and decreased nitric oxide release. In vivo, the effects of high salt diet on endothelial ENaC expression and activity have varied depending on the animal model utilized. Our aim in the present study was to examine the role of endothelial ENaC in mediating vasorelaxation in the C57Bl/6 mouse strain. We utilized pressure myography to test the responsiveness of thoracodorsal arteries to acetylcholine in mice with increased sodium consumption both in the presence and absence of increased aldosterone. ENaC’s contribution was assessed with the use of the specific inhibitor amiloride. We found that while aldosterone had very little effect on ENaC's contribution to acetylcholine sensitivity, a high salt diet led to an amiloride‐dependent shift in the acetylcholine response of vessels. However, the direction of this shift was dependent on the length of high salt diet administration. Overall, our studies reveal that ENaC's role in the endothelium may be more complicated than previously thought. The channel does not simply inhibit nitric oxide generation, but instead helps preserve a homeostatic response.

## Graphical abstract

The influence of endothelial epithelial Na^+^ channel (ENaC) on acetylcholine‐dependent vasodilation of thoracodorsal arteries changes over time when mice are on a high salt diet. An initial enhancement evolves to an ENaC‐dependent blunting of the vasodilation.

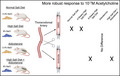

## 1 INTRODUCTION

The epithelial Na^+^ channel (ENaC) is a key mediator of Na^+^ flux across the apical membrane of specific, high resistance epithelia. Channel function is critical for extracellular fluid volume and blood pressure (BP) homeostasis, as it facilitates the reabsorption of the final 3%–5% of filtered Na^+^ in the aldosterone‐sensitive distal nephron (Kleyman & Eaton, 2020). Both gain‐ and loss‐of‐function mutations in the channel can lead to BP disorders, underscoring its importance (Levanovich et al., 2020). The channel's role in BP regulation has more recently been found to extend beyond the kidney, as its expression in non‐epithelial tissues, including dendritic cells, vascular smooth muscle, and endothelium influences vascular tone and BP (Barbaro et al., 2017; Mutchler et al., 2021; Tarjus et al., 2017; VanLandingham et al., 2009). In endothelial cells, ENaC activation has been shown to contribute to vascular dysfunction in the setting of high fat diet (Sowers et al., 2019; Xiong et al., 2020), atherosclerosis (Liang et al., 2018; Niu et al., 2021), aging (Paar et al., 2014), and high salt diet (HSD; Yang et al., 2020).

Endothelial ENaC facilitates dysfunction, in part, by limiting the generation of the gaseous vasodilator nitric oxide (NO; Kusche‐Vihrog et al., 2014). Increased expression of endothelial ENaC has been demonstrated to positively correlate with the density of the cortical actin cytoskeleton directly beneath the cell membrane (Jeggle et al., 2013). This increase in actin density renders endothelial cells less sensitive to shear stress, which decreases activation of endothelial nitric oxide synthase (eNOS), the enzyme responsible for NO production (Fels et al., 2012; Oberleithner et al., 2007). Decreased NO leads to vascular dysfunction as characterized by stunted relaxation and vessel stiffening that can eventually lead to hypertension.

Whereas renal ENaC is inhibited by increases in extracellular [Na^+^], endothelial ENaC has been proposed to exhibit a novel feedforward activation whereby high [Na^+^] increases ENaC expression and activity (Korte et al., 2014). However, studies utilizing isolated vessels and the ENaC inhibitor amiloride have shown that different animal models may vary in their vascular ENaC response to extracellular Na^+^. In Sprague‐Dawley rats on a control salt diet, amiloride pre‐treatment of isolated arteries slightly increased acetylcholine (Ach) sensitivity (Liu et al., 2015). When these rats were switched to a HSD for 3 weeks, Ach sensitivity increased while ENaC expression decreased, and the vessels no longer showed an amiloride‐sensitive component of vasodilation (Liu et al., 2015). When the same experiments were repeated in Dahl salt‐sensitive rats, a HSD caused a paradoxical increase in plasma aldosterone that stimulated ENaC expression in the endothelium leading to decreased Ach sensitivity that was ameliorated with amiloride pretreatment (Wang et al., 2018).

Given these strain‐specific responses of endothelial ENaC to high salt in rats mentioned above, the aim of this project was to examine the response of endothelial ENaC to HSD in the C57Bl/6 mouse strain, as this line is frequently used for genetic manipulations. We examined whether aldosterone altered this response given its role in modulating ENaC expression in other tissues (Masilamani et al., 1999) and the observation that salt and aldosterone may act synergistically in the endothelium to promote ENaC expression (Korte et al., 2014). We analyzed Ach sensitivity in thoracodorsal arteries from mice receiving a HSD, with or without aldosterone, both in the presence and absence of luminal pre‐treatment with a low concentration (5 µM) of the specific ENaC inhibitor amiloride. We hypothesized that amiloride would not cause a change in Ach sensitivity with HSD alone given its absence of effect in Sprague‐Dawley rats on HSD (Liu et al., 2015). We further hypothesized that ENaC would promote vascular dysfunction in mice receiving both aldosterone and HSD, which would result in increased Ach‐mediated dilation when amiloride is present in these vessels.

## 2 METHODS

### 2.1 Animals

Male, C57Bl/6J male mice, aged 9–12 weeks, were purchased from Jackson Laboratories (Bar Harbor, ME) and assigned to one of four groups: normal salt diet (NSD), HSD, aldosterone (aldo), or HSD and aldosterone (HSD + aldo). NSD animals were fed a standard chow containing 0.4% NaCl, HSD animals were given a diet containing 8% NaCl (TD.92012) for 4 or 8 weeks, and aldosterone mice were implanted with a subcutaneous minipump (model 2002, Alzet, Cupertino, CA) to release aldosterone at a rate of 240 μg/kg/day (Pruthi et al., 2014) for 2 weeks. HSD+aldosterone animals received HSD alone for the first 2 weeks at which point they had a minipump inserted to receive a combination of HSD with aldosterone administration for their final 2 weeks of treatment. Minipumps were inserted via a small incision behind the neck which allowed for the creation of a subcutaneous pocket extending to the right flank. Mice were anesthetized with isoflurane for the procedure. Aldosterone (Sigma) was given in a 5% EtOH solution. Mice were sacrificed under isoflurane anesthesia with terminal cardiac puncture and exsanguination with blood collected into a heparinized needle. [Na^+^] in whole blood was measured by an iSTAT analyzer (Abott Laboratories) and plasma separated via centrifugation to be assayed on a commercially available aldosterone ELISA according to the manufacturer's directions (Enzo Life Sciences). All animal protocols were approved by the Institutional Animal Care and Use Committee at the University of Pittsburgh.

### 2.2 Cell Culture

Human umbilical vein endothelial cells (HUVEC) were purchased from PromoCell and grown in Endothelial Cell Growth Medium 2 supplemented with Growth Medium 2 Supplement Pack (PromoCell) and 5% fetal calf serum (FCS). Cells were maintained on gelatin‐coated plates until they were harvested into Trizol for RNA extraction according to the manufacturer's instructions (Ambion), and cDNA was synthesized with the iScript cDNA synthesis kit (BioRad Laboratories) with 1 μg of RNA being added per cDNA reaction and 1 µl of cDNA added per RT‐PCR reaction. The following primers were used for identification of human ENaC subunits: α‐subunit‐1 forward 5′‐AATTCGGCCTGCTTTTCGGA‐3′, reverse 5′‐ACAGGTCAAAGAGCGTCTGC‐3′; α‐subunit‐2 forward 5′‐CAGCTTGCGGGACAACAAC‐3′, reverse 5′‐CA GGCGAAGATGAAGTTGCC‐3′; α‐subunit‐3 forward 5′‐GTGTGGCTGTGCCTACATCT‐3′, reverse 5′‐AGAGA GCTGGTAGCTGGTCA‐3′; β‐subunit‐1 forward 5′‐CAGT GCTACCCAGGCATTGA‐3′, reverse 5′‐CATGGCGTAG ATGCCCTCAT‐3′; β‐subunit‐2 forward 5′‐ACCGGAACT TCACGTCCATC‐3′, reverse 5′‐TTTGGACGGGGACCTC AGAA‐3′; β‐subunit‐3 forward 5′‐GTGACTACAACACG ACCTACTC‐3′, reverse 5′‐TTCCTGCTCAGGGTGATAT TG‐3′; γ‐subunit‐1 forward 5′‐CTTGGACCCTTTGGAAC CGA‐3′, reverse 5′‐GCAGTCAGTGTGAACCCGAT‐3′; γ‐subunit‐2 forward 5′ TCGGCAGGATGAGTATCCCT‐3′, reverse 5′‐ AGGCAGATCTGGAGCGAGTA‐3′; γ‐subunit‐3 forward 5′‐ AGAAGTGGTTGCTGCCTGTT‐3′, reverse 5′‐ GCCACCGAAGTTGGACAGAA‐3′. Conditions for PCR are given below.

### 2.3 Magnetic cell separation

Endothelial cells were isolated from mouse heart, lung, brain, and kidney using the Dynabead FlowComp Flexi Kit (Invitrogen). A CD31 antibody (553370, BD Biosciences) was biotinylated according to the manufacturer's directions for the supplied DSB‐X Biotin Protein Labeling Kit. Organs were minced and allowed to incubate with 3% collagenase in PBS at 37°C with shaking for 30 min. After passage through a cell strainer and centrifugation, cell pellets were resuspended in 2 ml of ammonium‐chloride‐potassium lysis buffer to remove red blood cells. After removal of the buffer, the cells were resuspended in 500 μl 2% FCS in phosphate buffered saline (PBS) and ECs were isolated according to the Dynabead FlowComp Flexi Kit published protocol. Endothelial isolates had RNA extracted using the standard Trizol protocol (Ambion), cDNA synthesized using the SuperScript III First‐Strand Synthesis SuperMix (Invitrogen) with 1μg of RNA being added per cDNA reaction. Reactions for RT‐PCR contained 1 μl of cDNA, 10 μl GoTaq Green Master Mix (Promega), and 0.5 μM forward and reverse primers. Reactions were cycled through the following: 95°C for 3 min (one cycle); 95°C for 20 s, 60°C for 30 s, 72°C for 40 s (50 cycles); 72°C for 5 min (one cycle). Mouse ENaC primers were as follows: α‐subunit forward 5′‐TGGATGCCGTGAGAGAATGG‐3′, reverse 5′‐ATGGGGTGGTGGAACTGAGA‐3′; β‐subunit forward 5′‐CACCACCTTAGCTGCCATCA‐3′, reverse 5′‐CCCCTCACAGATGATGCGTT‐3′; γ‐subunit forward 5′‐GCCGTGACCCTTCAGTTCAG‐3′, reverse 5′‐CTTAATGGTCGGTGCCTGGG‐3′.

### 2.4 Pressure myography

Thoracodorsal arteries (TDA) were isolated, cannulated, and pressurized in a Danish MyoTechnology (DMT) pressure myograph following procedures outlined in the literature (Billaud et al., 2012). TDAs were carefully excised by making an incision in the skin above the scapula to expose the underlying muscle and fat was cleared from the artery. Vessels were kept in cold Krebs‐Hepes buffer (NaCl 118.4 mM, KCl 4.7 mM, MgSO_4_ 1.2 mM, NaHCO_3_ 4 mM, KH_2_PO_4_ 1.2 mM, CaCl_2_ 2 mM, Hepes 10 mM, glucose 6 mM). Vessel segments were cannulated at each end and secured with 11–0 silk suture. A small amount of flow was allowed through the lumen to clear any remaining blood. Vessels were perfused with a warmed 1% BSA supplemented Krebs‐HEPES buffer (pH =7.4) and allowed to equilibrate for 30 min in a 37°C circulating bath at a pressure of 80 mmHg. For amiloride treated vessels, the BSA containing perfusate had amiloride added at a final concentration of 5 μM while control vessels had an equivalent amount of DMSO added. Vessel lumens were perfused with these solutions prior to the 30 min equilibration period. Vessels were preconstricted with phenylephrine (PE; 10^−5^ M) for 15 min which was added to the bath. Increasing doses of acetylcholine (Ach; 10^−9^ M through 10^−4^ M) or sodium nitroprusside (SNP; 10^−10^ M through 10^−3^ M) were added to the bath and allowed to circulate for 5 min. Both the highest vessel diameter achieved with a specific Ach dose and the diameter at the end of a 5 min perfusion with a specific Ach dose were measured. KCl (40 mM) was added at the end of the experiment to ensure equal contraction. Vessels that did not respond with at least 70% constriction to KCl were excluded from the analysis. The maximum vessel diameter was measured by incubating the vessel in a calcium‐free Krebs solution supplemented with ethylene glycol tetraacetic acid (EGTA, 1 mM) and sodium nitroprusside (SNP 10 μM). If the diameter reached under these conditions was not higher than the initial diameter measured after equilibration, the vessel was also excluded from the analysis. Quantification of vessel diameter was performed using DMT vessel acquisition software and data from vasodilation experiments are expressed as the percentage of maximal dilation calculated as ((diameter at time point) − (diameter at end of preconstriction))/((diameter with EGTA) − (diameter at end of preconstriction)) ×100. Data were calculated using the maximal diameter reached in a dose unless otherwise noted. All chemicals were obtained from Sigma.

### 2.5 Statistical analysis

Data are presented as mean ± SD unless otherwise noted. All statistics were performed in GraphPad Prism (GraphPad Software). Dose response curves were analyzed by two‐way ANOVA with Sidak's multiple comparisons test. Ach dose response curves are shown with a nonlinear curve of the log(agonist) versus response (three parameters) which was used to determine logEC_50_. Bar graphs were analyzed using *t*‐test or one‐way ANOVA with Tukey's multiple comparisons test. Significance is identified as: *p ≤ 0.05, **p ≤ 0.01, ***p ≤ 0.001, or ****p ≤ 0.0001. All *n* values denote a single vessel from a single animal. Multiple vessels from one animal were not included in the same group unless otherwise noted.

## 3 RESULTS

### 3.1 HUVECs and isolated murine endothelial cells express ENaC subunits

**FIGURE 1:**
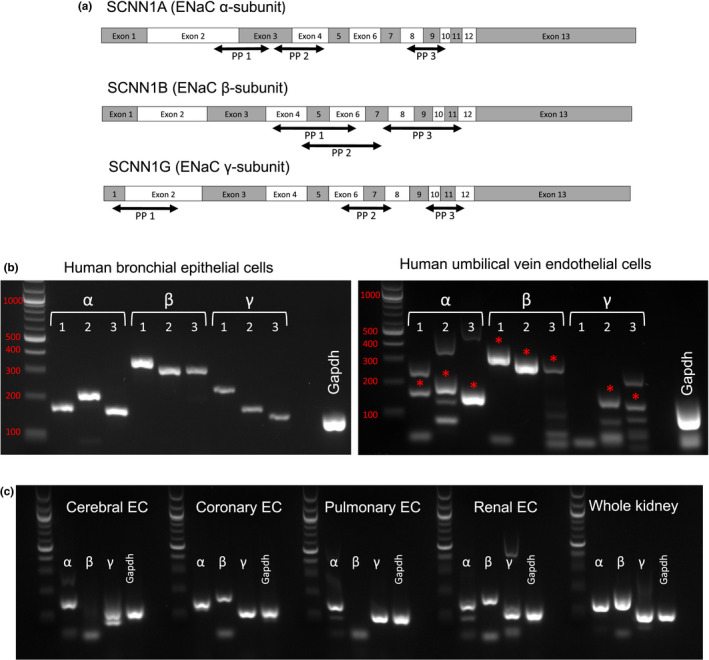
Expression of epithelial Na^+^ channel (ENaC) in cultured human and primary murine endothelial cells. (a) Locations of sequences recognized by three separate primer pairs to the human α‐, β‐, and γ‐subunits of ENaC. (b) Primers were validated on cDNA prepared from primary human bronchial epithelial cells to confirm size. (c) Results of RT‐PCR performed on HUVECs with the proper ENaC band identified by red asterisk. (d) Isolated endothelial cells (ECs) from murine brain, heart, lung and kidney were examined for ENaC subunit mRNAs using one primar pair. Bands show expression of all three subunits in the coronary and renal EC populations. Cerebral and pulmonary beds expressed the α‐ and γ‐subunits, but lacked a clear β‐subunit band

A recent report was unable to reliably identify ENaC transcripts in cultured endothelial cells and isolated vessels (Ydegaard et al., 2019). Therefore, we first used RT‐PCR to examine the expression of ENaC α, β, and γ subunit message in HUVECs and isolated mouse endothelial cells to identify whether the investigation of amiloride sensitivity in endothelium was warranted. In HUVECs, we used three separate primer pairs for each subunit, localized to different regions of the genes (Figure 1a). The efficacy of these primers was ensured by performing RT‐PCR on mRNA isolated from primary human bronchial epithelial (HBE) cells, known to express ENaC (Figure 1b). HUVECs did display products of the proper size (Figure 1c, correct bands denoted by asterisks), and these were verified to be ENaC through sequencing. However, the HUVEC bands did not have the same intensity as the HBEs, and the presence of presumably non‐specific HUVEC PCR products were noted, suggesting lower expression of the target sequence in the endothelial cells. Primer pair one for the γ subunit did not give a PCR product in three separate reactions from the same isolated cDNA, suggesting this region may be altered in endothelial cells.

To examine the expression of ENaC subunits in murine endothelium, CD31 labeled magnetic beads were used to separate ECs from the heart, brain, kidney, and lung of C57Bl/6 male mice on a control sodium diet (0.4% NaCl). Using RT‐PCR, we probed for the presence of the ENaC subunits in each of these vascular beds. ENaC expression in whole kidney was performed as a positive control. Products for the α and γ subunits were detected in all EC populations (Figure 1d). A product for the β subunit was observed in the coronary and renal ECs, but it was not detected in cerebral and pulmonary ECs, suggesting that differences in β subunit expression may exist across vascular beds under non‐stimulated conditions.

### 3.2 Amiloride alters Ach‐responsiveness in TDAs from mice receiving a HSD with or without aldosterone

ENaC expression in isolated ECs has been shown to be stimulated by both aldosterone and high [Na^+^] (Jeggle et al., 2016; Kusche‐Vihrog et al., 2008). Therefore, we studied the effects of an ENaC inhibitor on vessels from mice treated in vivo with these stimuli alone or in combination. We examined the Ach responsiveness of TDAs isolated from the normal salt diet (NSD) fed mice as well as from mice receiving HSD, aldosterone, or HSD and aldosterone. TDAs from mice were split in two. Vessel lumens were perfused with a DMSO containing control solution or with a solution containing the ENaC inhibitor amiloride (5 μM, a concentration specific for ENaC (Kleyman & Cragoe, 1988)) prior to the 30 min equilibration period.

**FIGURE 2:**
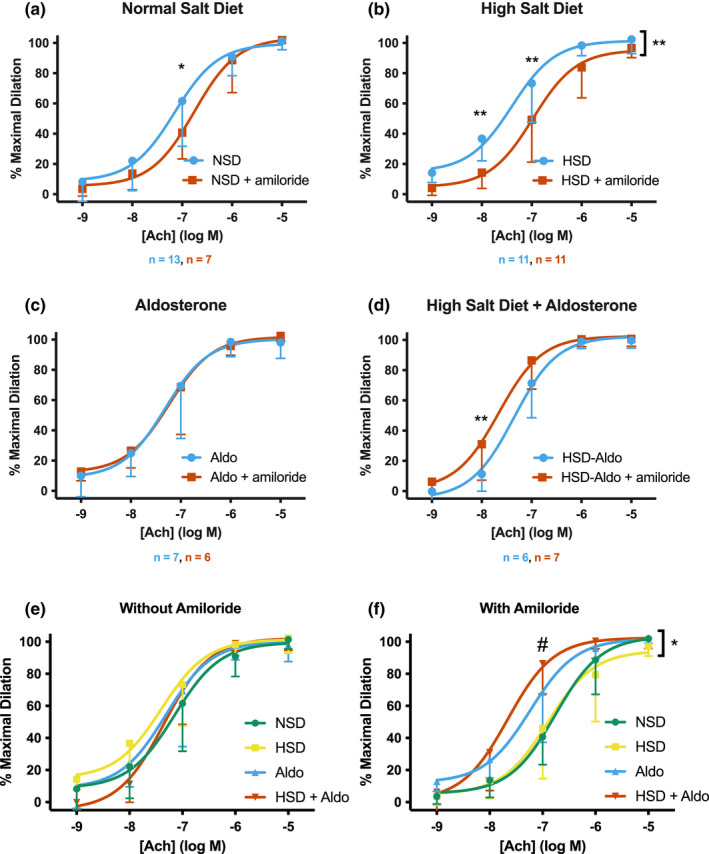
Effects of amiloride on Ach‐sensitivity in Thoracodorsal arteries (TDAs) from NSD mice or mice treated with 4‐weeks high salt diet (HSD) and/or aldosterone. Ach dose response curves were generated in TDAs from animals given a normal salt diet (a), 4‐weeks HSD (b), 2‐weeks aldosterone (c) or a combination treatment (d). Vessels treated with DMSO vehicle are shown in blue while vessels treated with luminal 5 μM amiloride are shown in red. The number of vessels tested from each condition are shown below the graphs. LogEC_50_ ± SEM for each curve are listed in Table 1. For *n* values, each vessel within the same treatment group is from a unique animal. Curves without amiloride pre‐treatment (e) or with amiloride pre‐treatment (f) from (a)–(d) are overlaid for comparison. All curves shown as mean ± SD with overlay of a nonlinear fit. Curves were analyzed for significance by two‐way ANOVA with multiple comparisons. *p ≤ 0.05, **p ≤ 0.01, ***p ≤ 0.001, or ****p ≤ 0.0001. A bracket with asterisk indicates a significant difference with treatment as the source of variation. In (f), # indicates the following significance from multiple comparisons (*p* value in parantheses): NSD versus aldo (0.02), NSD versus HSD + aldo (<0.0001), HSD versus aldo (0.0486), HSD versus HSD+aldo (<0.0001) (see Table 2).

Following preconstriction with PE (10^−5^ M), vessels from NSD animals responsed to increasing concentrations of Ach with dose‐dependent dilation, reaching a plateau at an [Ach] of 10^−6^ M. Amiloride pre‐treatment of vessels from NSD animals led to a significant decrease in vessel diameter at an [Ach] of 10^−7^ M, suggesting that under baseline conditions ENaC moderately enhances Ach‐dependent vasodilation in TDAs (Figure 2a). However, significant differences between the EC_50_s were not observed (see Table 1). When animals were fed a HSD for 4 weeks, the amiloride‐dependent shift in the Ach response was more apparent, with significant reductions in vessel diameter at an [Ach] of 10^−8^ and 10^−7^ M with amiloride pre‐treatment (Figure 2b). Additionally, the EC_50_s of the control‐ and amiloride‐treated curves were significantly different in vessels from HSD animals (Table 1). Amiloride had no effect on Ach‐sensitivity in vessels from mice treated with aldosterone alone (Figure 2c). However, when the aldosterone was given with HSD, amiloride pre‐treatment led to a significant increase in the vessel diameter at an [Ach] of 10^−8^ M, opposite to what was observed in the NSD and HSD vessels (Figure 2d).

**TABLE 1:** logEC_50_ values for Ach dose response curves with normal salt diet, high salt diet, aldosterone, or high salt diet and aldosterone treatment. *N* values are included in parantheses next to the logEC_50_ value. Each *n* value denotes a vessel from an individual animal

Treatment	logEC_50_ control	logEC_50_ amiloride	*p* value	Avg vessel diameter (µm)	
				Control	Amiloride
Normal salt	−7.15 ± 0.12 (13)	−6.76 ± 0.12 (7)	0.17	202 ± 6.8	198 ± 4.7
High salt	−7.40 ± 0.12 (11)	−6.98 ± 0.12 (11)	0.0019 (**)	198 ± 7.2	202 ± 4.9
Aldosterone	−7.31 ± 0.18 (7)	−7.24 ± 0.16 (6)	0.86	208 ± 11.5	198 ± 5.9
High Salt + Aldosterone	−7.35 ± 0.12 (6)	−7.66 ± 0.15 (7)	0.14	199 ± 8.2	213 ± 3.1

** represents p ≤ 0.01

Curves from panels (a)–(d) were separated according to the presence or absence of amiloride to compare Ach sensitivities between treatments. When we examined Ach‐dependent vasodilation in the absence of amiloride, similar responses were noted in vessels from mice under all four experimental conditions (NSD, HSD, aldo, HSD+aldo, see Figure 2e) and the EC_50_s did not differ (Table 2). However, in the presence of amiloride, the Ach‐responses diverge, as ENaC inhibition enhanced vasodilation in NSD or HSD vessels but suppressed vasodilation from HSD+aldo vessels (Figure 2f). This suggests that ENaC has a role in maintaining a “normal” vasodilatory response under these four conditions, rather than simply inhibiting or promoting vasodilation.

**TABLE 2:** logEC_50_ values comparing treatments with and without amiloride in the lumen. *N* values are included in parantheses next to the logEC_50_ value. Each *n* value denotes a vessel from an individual animal

	Normal salt (NS)	High salt (HS)	Aldosterone (A)	High salt+Aldosterone (HSA)	*p* values
Without amiloride	−7.15 ± 0.12 (13)	−7.40 ± 0.12 (11)	−7.31 ± 0.18 (7)	−7.35 ± 0.12 (6)	NS vs HS: 0.22 NS vs A: 0.90 NS vs HSA: 0.99 HS vs A: 0.76 HS vs HSA: 0.33 A vs HSA: 0.90
With amiloride	−6.76 ± 0.12 (7)	−6.98 ± 0.12 (11)	−7.24 ± 0.16 (6)	−7.66 ± 0.15 (7)	NS vs HS: 0.98 NS vs A: 0.32 NS vs HSA: 0.09 HS vs A: 0.14 HS vs HSA: 0.03* A vs HSA: 0.94

**FIGURE 3:**
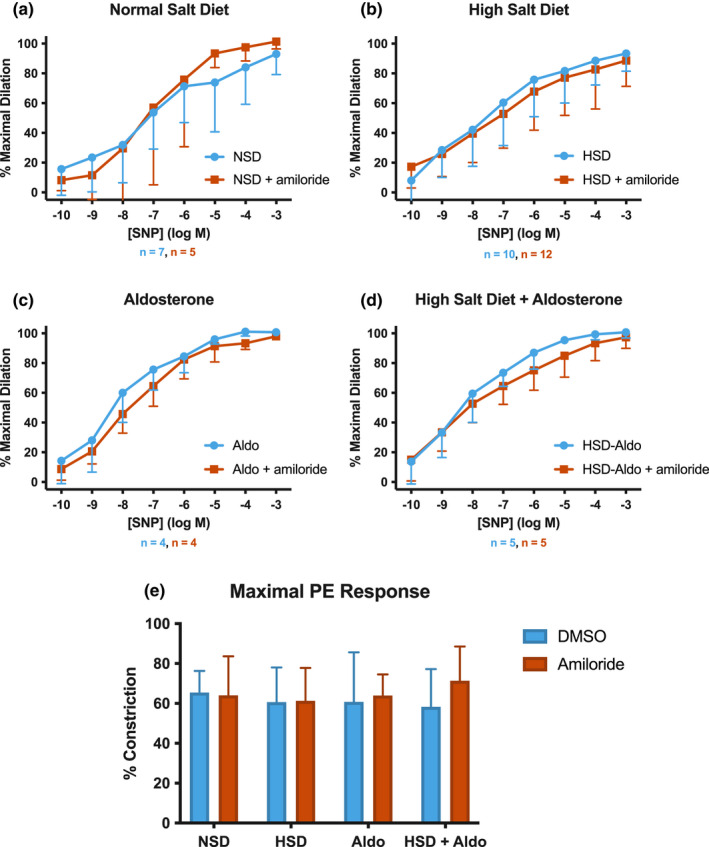
Amiloride pre‐treatment does not alter the response of thoracodorsal arteries (TDAs) to SNP or PE. SNP dose response curves were generated in TDAs from animals given a normal salt diet (a), 4‐weeks high salt diet (b), 2‐weeks of aldosterone (c) or a combination treatment (d). Vessels treated with DMSO vehicle are shown in blue while vessels treated with luminal 5 μM amiloride are shown in red. N value is shown beneath each graph in the corresponding color with each vessel in a group being obtained from a separate animal. All points are shown as mean ± SD. (e) The maximal constriction during the 15‐min phenylephrine constriction was measured and calculated as (1 − (constricted diameter)/(initial diameter)) × 100. No significant difference was noted by one‐way ANOVA

To separate endothelial dysfunction from an inability of the smooth muscle to respond to NO, the vasodilatory response of TDAs to increasing doses of the NO donor sodium nitroprusside (SNP) was also assessed. There were no significant differences in the response to SNP in TDAs, with or without amiloride in the vessel lumen, among the four groups (Figure 3a–d). In addition, the peak PE response, measured as the smallest diameter obtained during the 15‐min preconstriction period, did not differ among the four treatment groups with or without amiloride (Figure 3e) suggesting there were no differences in the contractile response of the TDAs. Additionally, the vessel diameter was not altered by treatment or the presence of amiloride (Table 1). Together, this suggests differences in Ach‐sensitivity are due to changes in the endothelium.

### 3.3 Length of HSD treatment affects the vessel response to Ach

Our results indicating ENaC improved Ach sensitivity in mice receiving a HSD for 4 weeks were surprising given previously published studies (Liu et al., 2015; Wang et al., 2018; Yang et al., 2020). However, we noted the vasodilatory response to increasing concentrations of Ach in the presence of amiloride was quite variable between animals. Vessel responses are separated into two distinct groups.

**FIGURE 4:**
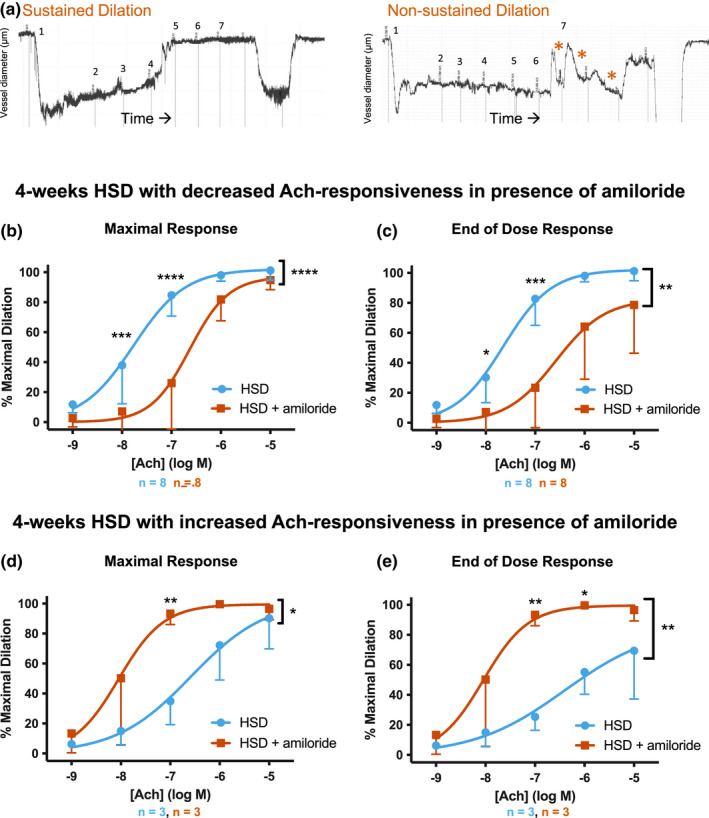
Thoracodorsal arteries (TDAs) from mice on 4‐weeks high salt diet (HSD) display two distinct responses. (a) Representative traces from a vessel with sustained dilation (left) or non‐sustained dilation (right). The *x*‐axis represents time while the the y‐axis represents vessel diameter. On both traces, number 1 indicates the addition of PE, while 2–7 indicate the addition of increasing doses of Ach. In the right trace, red asterisks indicate doses of Ach that resulted in non‐sustained dilation. (b–e) TDAs from mice treated with 4‐weeks HSD were separated according to whether they had blunted Ach‐sensitivity with amiloride pre‐treatment (b, c) or enhanced response with amiloride (d, e). Curves were generated based on the maximal diameter achieved during the dose (b, d) or the diameter of the vessel at the end of the incubation with each dose of Ach (c, e). All curves shown as mean ± SD with overlay of a nonlinear fit. The number of vessels tested from each condition are shown below the graphs. LogEC_50_ ± SD for each curve are listed in Table 3. Curves were analyzed for significance by two‐way ANOVA with multiple comparisons. *p ≤ 0.05, **p ≤ 0.01, ***p ≤ 0.001, or ****p ≤ 0.0001. A bracket with asterisk indicates a significant difference with treatment as the source of variation

Whereas the majority of TDAs taken from our untreated, NSD mice react to a dose of Ach by dilating and remaining at this new diameter (“sustained dilation”, illustrated in Figure 4a, left panel), we observed that some of our vessels from our different treatments dilated in response to Ach but rapidly constricted again (“non‐sustained dilation”, Figure 4a, right panel, indicated by red astericks). In particular, we noted two distinct subgroups in the 4 weeks HSD treatment group: (i) Animals that had normal Ach sensitivity in control vessels displayed a blunted vasodilatory response and non‐sustained dilation in the presence of amiloride, or (ii) animals had decreased Ach sensitivity and non‐sustained dilation in control vessels that was improved in the presence of amiloride. We separated these two groups, using the response at 10^−7^ M Ach between the control treated and amiloride treated vessel from the same animal for comparison.

The majority of 4 weeks HSD‐fed mice (*n* = 8) exhibited the first type of response with blunted Ach sensitivity in the presence of luminal amiloride, consistent with results presented in Figure 2. Some amiloride treated vessels in this group (5 out of 8) were unable to sustain dilation at higher concentrations of Ach, which is illustrated by the difference in the dose response curves when the diameter was measured as the maximal dilation within a specific [Ach] (Figure 4b), versus the diameter at the end of the 5‐min treatment with the same [Ach] (Figure 4c). Amiloride pre‐treatment of these TDAs also prevented a full dilatory response at the completion of Ach treatment, as the TDAs did not reach 100% maximal diameter (Figure 4c).

A minority of the 4 weeks HSD cohort (*n* = 3) fell into the second subgroup that responded in the opposite manner. In these animals, TDAs exhibited enhanced Ach sensitivity in the presence of amiloride, suggesting ENaC‐dependent endothelial dysfunction. In the absence of amiloride, vessels from these three HSD mice were also unable to sustain dilation, as noted by the difference between maximal response versus the end‐of‐dose response (Figure 4d,e). Furthermore, in the absence of amiloride these TDAs did not reach 100% of their maximal dilation with the higher doses of Ach (Figure 4e). In all three of these animals, amiloride pre‐treatment restored Ach‐sensitivity, shifting the dose response curve to the left (Figure 4d,e). Amiloride also allowed for sustained dilation during the 5‐min incubation, and restored the full vasodilatory capacity of the vessels.

**FIGURE 5:**
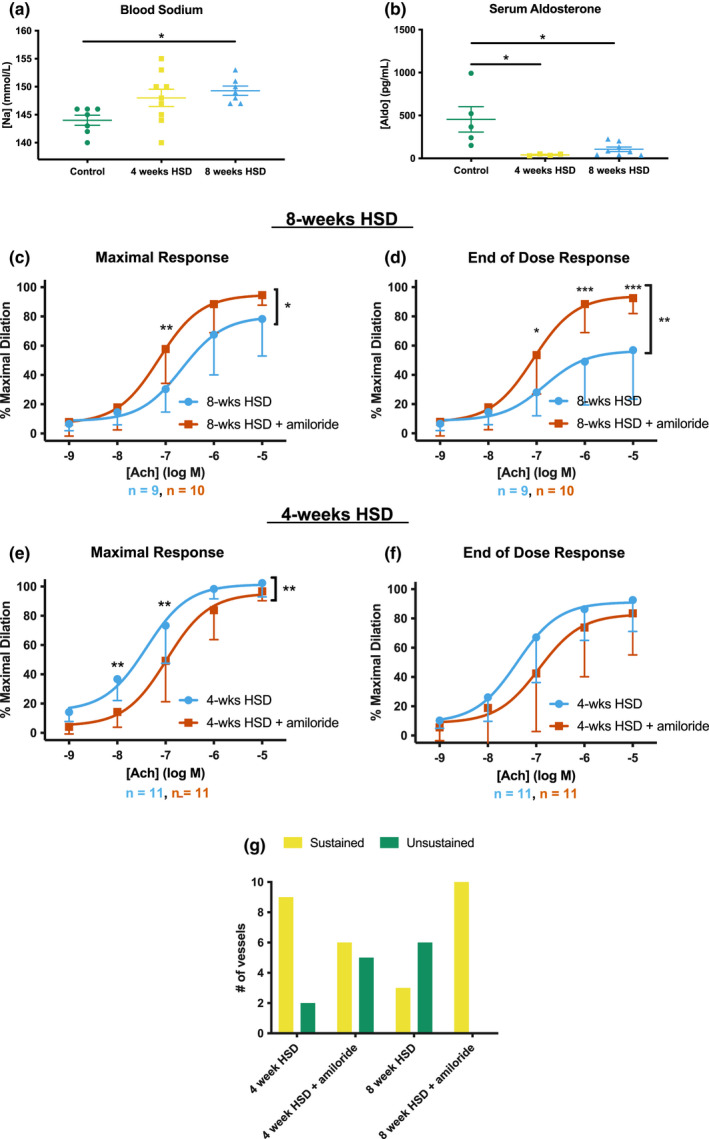
Thoracodorsal arteries (TDAs) from mice on 8‐weeks high salt diet (HSD) exhibit impaired Ach‐responsiveness that is ameliorated with amiloride. (a) Whole blood [Na^+^] was measured from mice on normal salt diet, 4‐weeks HSD, or 8‐weeks HSD using an iSTAT analyzer. [Na^+^] was significantly higher in mice on 8‐weeks HSD, compared to normal salt diet animals as assessed by one‐way ANOVA (b) Serum [Aldosterone] was significantly lower in mice on 4‐ or 8‐weeks HSD, compared to normal salt diet. (c, d) Ach dose response curves were generated in TDAs from mice on 8‐weeks HSD, displayed as both the maximal response within a dose (c) and the diameter at the end of a dose (d). (e, f) For comparison, the aggregate Ach response of TDAs taken from mice on 4‐weeks HSD, displayed as maximal (e) and end of dose (f) diameters. (h) Quantification of the number of TDAs within each treatment protocol that responded with a sustained or a non‐sustained dilation, as illustrated by the curves in Figure 4a and defined within the text. For (c)–(d), the number of vessels tested from each condition are shown below the graphs. LogEC_50_ ± SD for each curve are listed in Table 3. Data in (a) and (b) were analyzed by one‐way ANOVA and dose response curves were analyzed by two‐way ANOVA. *p ≤ 0.05, **p ≤ 0.01, ***p ≤ 0.001, or ****p ≤ 0.0001. A bracket with asterisk indicates a significant difference with treatment as the source of variation

The presence of two distinct responses to Ach when mice were on 4 weeks HSD led us to hypothesize that 4 weeks of HSD reflects a dynamic time point when compensatory responses that maintain dilation start to become detrimental. To test this hypothesis, we lengthened our HSD treatment to 8 weeks to determine if the Ach response evolved to one phenotype. With the increased time on HSD, we noted a gradual increase in the blood [Na^+^] (Figure 5a; NSD: 144 ± 1 mM (*n* = 7); 4‐weeks HSD: 148 ± 2 mM (*n* = 9); 8‐weeks HSD: 149 ± 1 mM (*n* = 7), NSD versus 8‐weeks HSD, *p* < 0.05, one‐way ANOVA with Tukey's multiple comparisons test). Plasma aldosterone levels were suppressed in mice on HSD, as expected (NSD: 454 ± 148 pg/ml (*n* = 5); 4‐weeks HSD: 41 ± 6 pg/ml (*n* = 4), 8‐weeks HSD 106 ± 26 pg/ml (*n* = 8), NSD versus 4‐ and 8‐weeks HSD, *p* < 0.05, one‐way ANOVA with Tukey's multiple comparisons test; Figure 5b). We again tested the sensitivity of TDAs from mice receiving 8‐weeks HSD to Ach. As above, results are shown as “maximal response” and “end of dose response,” and dose response curves from all mice in the 4‐weeks HSD cohort are shown in Figure 5e,f for comparison. TDAs from mice on 8‐weeks HSD exhibited a failure to fully dilate to the higher doses of Ach (Figure 5c,d) as well as an inability to sustain dilation over 5 min at higher doses of Ach. The addition of amiloride in the lumen restored Ach sensitivity and allowed for sustained dilation (Figure 5f). These data suggest that ENaC impairs the vasodilatory response of TDAs to Ach when mice are on HSD for 8 weeks. Comparisons of EC_50_s are shown in Table 3.

**TABLE 3:** logEC_50_ values for animals receiving 4‐ or 8‐weeks of high salt diet (HSD) treatment. *N* values are included in parantheses next to the logEC_50_ value. Each *n* value denotes a vessel from an individual animal

	logEC_50_ control	logEC_50_ amiloride	*p* value
*4‐weeks HSD (all vessels)*			
Maximal response	−7.40 ± 0.12 (11)	−6.98 ± 0.12 (11)	0.0019**
End of dose response	−7.39 ± 0.18 (11)	−6.93 ± 0.27 (11)	0.19
*4‐weeks HSD with decreased Ach‐responsiveness in presence of amiloride*			
Maximal response	−7.77 ± 0.14 (8)	−6.63 ± 0.27 (8)	0.002**
End of dose response	−7.64 ± 0.13 (8)	−6.58 ± 0.38 (8)	0.001**
*4‐weeks HSD with decreased Ach‐responsiveness in presence of amiloride*			
Maximal response	−6.58 ± 0.22 (3)	−8.04 ± 0.44 (3)	0.04*
End of dose response	−6.41 ± 0.26 (3)	−8.04 ± 0.44 (3)	0.02*
*8‐weeks HSD (all vessels)*			
Maximal response	−6.66 ± 0.21 (9)	−7.12 ± 0.13 (10)	0.03*
End of dose response	−6.83 ± 0.34 (9)	−7.07 ± 0.14 (10)	0.007*

* represents p ≤ 0.05. ** represents p ≤ 0.01.

We quantified the number of sustained versus unsustained dilators within each treatment group (Figure 5g). A non‐sustained Ach response was defined as any TDA where the difference between the maximal diameter and end diameter in at least two Ach doses was ≥10%. The majority of TDAs from mice on 4‐weeks HSD exhibited a sustained dilatory response to Ach (82% [9 of 11]). However, when amiloride was added to the lumen, this number decreased to 55% (6 of 11). This trend was reversed in TDAs from mice on 8‐weeks HSD, where only 33% of control vessels (3 of 9) showed sustained dilation. When amiloride was added to the vessel lumen, all 10 TDAs from mice on 8‐weeks HSD exhibited sustained dilation in response to Ach. Together, these data illustrate that amiloride has distinct effects on Ach sensitivity and vasodilatory behavior in TDAs from mice on a NSD, 4 weeks of HSD, or 8 weeks of HSD.

**FIGURE 6:**
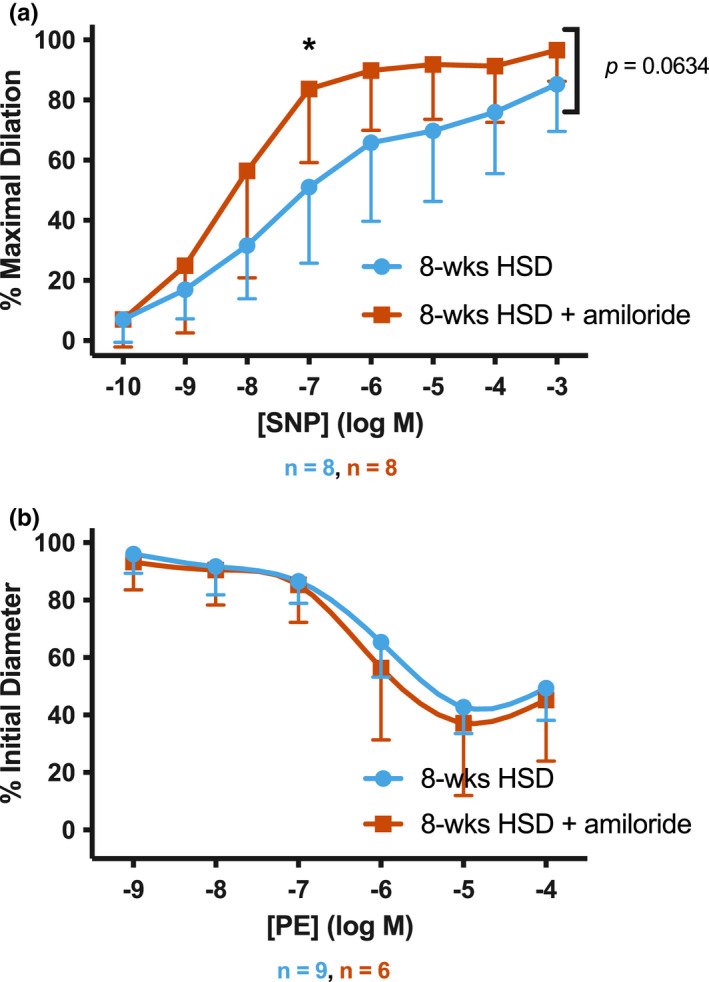
Sodium nitroprusside (SNP) and phenylephrine (PE) dose responses from 8‐weeks high salt diet (HSD). (a) SNP dose response curves and (b) PE dose response curves were generated in TDAs from mice given 8‐weeks HSD. Data are displayed as mean ± SD. Curves were analyzed for significant difference by two‐way ANOVA

Finally, we examined the response of TDAs from mice on 8‐weeks HSD to SNP and PE in the absence of presence of luminal amiloride. Lower concentrations of SNP were required to achieve a dilatory response in the presence of amiloride (Figure 6a), consistent with the role of ENaC in dampening the responsiveness to NO. However, amiloride did not alter the response to PE (Figure 6b), suggesting ENaC does not play a role in sensitizing these vessels to this vasoconstrictor.

## 4 DISCUSSION

Our data support previously published reports of ENaC expression in both cultured human ECs and freshly isolated murine ECs (Downs et al., 2018; Kusche‐Vihrog et al., 2008). We did not perform quantitative PCR on these samples, however, our results suggest lower levels of expression in the endothelium as compared to the kidney or lung as we saw the presence of non‐specific bands and decreased density of the bands of interest. Our observations are consistent with ENaC’s role in mediating transepithelial Na^+^ currents in kidney and lung where the higher levels of expression are required for bulk Na^+^ transport. In contrast, lower levels of channel expression and Na^+^ currents are likely needed to facilitate changes to the Na^+^ microenvironment in endothelia. It has been suggested that these small ENaC‐dependent increases in intracellular [Na^+^] in ECs enhance the stability of polymerized actin to increase the density of the cortical actin cytoskeleton which causes endothelial stiffness and reduces the activity of eNOS (Fels & Kusche‐Vihrog, 2020; Oda et al., 2001). In addition, Na^+^ influx in ECs inhibits the transport of l‐arginine, the precursor to NO, which would also act to limit the activity of eNOS (Guo et al., 2016). Beyond simply acting as a conduit for Na^+^ transport, ENaC also has mechanosensitive properties that may allow it to function as a sensor of shear stress in ECs, which could couple it to downstream signaling mechanisms (Ashley et al., 2018; Knoepp et al., 2020). This signaling role would potentially require fewer channels to be expressed, as amplification of signaling occurs downstream.

The paucity of β subunit expression in ECs from mouse cerebral and pulmonary vascular beds suggests that some ECs may express αγ channels rather than canonical αβγ channels. The existence of non‐canonical channels consisting of an α subunit coupled with the acid sensing ion channel 1a (ASIC1a) has been proposed in pulmonary ECs (Czikora et al., 2017). Additionally, mice lacking the α‐subunit of ENaC in ECs have a different response to renal ischemia‐reperfusion compared to mice that lack the γ subunit in ECs, supporting the idea that ENaC with different subunit compositions have different functional effects in endothelia (Mutchler et al., 2021; Tarjus et al., 2019). Given the low ENaC mRNA levels in ECs, further analyses are needed to ascertain ENaC subunit composition in specific endothelial beds, and additional work is needed to understand what other proteins ENaC subunits may be multimerizing with. We attempted to measure changes in ENaC mRNA expression in TDAs. However, given the small amount of tissue and low degree of expression, we were unable to detect ENaC message by qPCR, or ENaC protein expression by immunofluorescence microscopy. Further work is therefore needed to understand whether salt is changing ENaC expression in vivo in TDAs or in other endothelial beds in mice.

ENaC in epithelial tissues displays inhibition by high extracellular [Na^+^], termed “Na^+^ self‐inhibition” (Sheng et al., 2006). This response allows the channel to quickly adapt to changes in the extracellular [Na^+^]. However, in endothelia ENaC expression has been shown to increase in response to an increase in extracellular [Na^+^] (Korte et al., 2014). Additionally, it has been shown to be transcriptionally regulated by the hormone aldosterone. However, this regulation has not been well studied in animals. Therefore, we created our model to explore the roles of HSD, aldosterone, or the combination of HSD and aldosterone to understand ENaC's role in promoting vascular dysfunction under these different conditions. We found that all ex vivo TDAs from our four groups of mice (NSD, 4‐weeks HSD, aldosterone, and HSD+aldosterone) had similar responses to Ach (Figure 2e). This was surprising, as previous work suggested that these stimuli are detrimental to vascular health (Fan et al., 2008; Galmiche et al., 2014). This lack of dysfunction may be due to our utilization of the thoracodorsal artery rather than the more commonly utilized mesenteric artery (Bridges et al., 2011). Interestingly, ENaC inhibition through amiloride pre‐treatment of the vessels caused a separation in the Ach dose response curves, revealing ENaC’s potential for facilitating a “normal” response to Ach (Figure 2f). From the separation of these curves, it appeared ENaC enhanced the Ach sensitivity of TDAs from NSD animals and the majority of mice on 4‐weeks HSD (Figures 2 and 4), which is contrary to the idea that endothelial ENaC limits NO generation (Kusche‐Vihrog et al., 2014). The only condition where ENaC slightly decreased Ach sensitivity was in animals given HSD + aldosterone, although not to the degree we hypothesized. While aldosterone enhances ENaC expression in many specific cell types, including ECs, we were surprised to find that amiloride did not affect the TDA response to Ach in vessels obtained from mice receiving aldosterone alone. This could reflect differences in ENaC’s contribution to endothelial function in different vascular beds, as aldosterone has been shown to promote endothelial stiffening and dysfunction in the aorta. These studies were performed in female mice, and sex differences in aldosterone sensitivity are known to exist (Jia et al., 2018) as well as differences in ENaC expression in the aldosterone‐sensitive distal nephron of male and female rats (Veiras et al., 2017). The response to and production of NO also varies between males and females (Reckelhoff et al., 1998), therefore, our studies need to be repeated in female animals in the future to determine whether sex‐specific differences exist.

While amiloride did not alter Ach sensitivity in the setting of a 2‐week aldosterone infusion, it displayed the largest effects in animals given HSD. While the majority of animals receiving 4‐weeks HSD showed improved Ach sensitivity and normal, sustained dilation without ENaC inhibition, animals on 8‐weeks HSD required ENaC inhibition to restore the Ach responsiveness. These results suggest that ENaC has a dynamic role in modulating the vasodilatory response to Ach, transitioning from enhancing to impairing the vessel's response to Ach depending on how long mice remain on a HSD. While the mechanism(s) underlying this transition in the amiloride‐sensitive Ach response is unclear, ENaC has been shown to localize to caveolae which serve as important signaling microdomains in ECs (Guo et al., 2016). Changes in ENaC's association with proteins in this highly dynamic space may explain this role reversal. Additionally, the ENaC‐dependent blunting of SNP sensitivity suggests that these changes may reflect pathways downstream of NO generation.

ENaC did not influence sensitivity to the NO donor SNP in TDAs from mice receiving control diet, 4‐weeks HSD, aldosterone alone, or HSD+aldosterone. However, ENaC did affect the SNP response in TDAs from mice on 8‐weeks HSD. Our results suggest that ENaC may influence the ability of the vessels to respond to NO when mice are on a long‐term HSD. The effects of amiloride on the sensitivity to SNP may be due to endothelial ENaC, however, we cannot exclude the possibility that amiloride given in the lumen of the vessel is crossing the endothelium and also inhibiting ENaC in vascular smooth muscle. Previous work has suggested that ENaC's role in the smooth muscle was primarily in mediating the myogenic response (Drummond et al., 2008; VanLandingham et al., 2009). Our observations could suggest a novel role for the channel in smooth muscle function in the setting of a long‐term HSD. As Ach can act at muscarinic receptors in VSM to cause constriction (Bolton & Lim, 1991), it is possible that some of the ENaC‐dependent effects we observed in TDAs in mice on 8 weeks HSD reflect changes in VSM signaling pathways. This could account for the non‐sustained dilation we observed in the 8‐weeks HSD vessels, and explain why amiloride pre‐treatment eliminates this inability of the vessels to remain fully dilated (Figure 5g).

Our studies have several limitations. We used a pharmacological inhibitor of ENaC rather than a genetic knockdown. The dose of amiloride utilized (5 μM) is specific for ENaC (Kleyman & Cragoe, 1988). NHE1 has an amiloride Ki that is more than 100‐fold higher than the Ki for ENaC (~0.1 µM) at a physiological [Na^+^] (Wangemann et al., 1996). Our RT‐PCR studies suggest that endothelial ENaCs are αβγ or αγ channels. The electrophysiological properties of ENaC in the endothelium are similar to channels found in renal epithelia (Liu et al., 2015), and αβγ or αγ channels exhibit similar amiloride sensitivities (McNicholas & Canessa, 1997). Previously published work showed there may be differences with acute, pharmacological inhibition of the channel versus genetic knockdown (Tarjus et al., 2017), and future work should explore the role of high salt and aldosterone in endothelial specific ENaC subunit knockout animals. Our studies focused on isolated TDAs due to their ease of dissection and the fact that one vessel could easily be split for studies with both control‐ and amiloride pre‐treatment. More work is needed to understand if other vascular beds, such as the mesentery or aorta, respond in the same manner with our various treatments. Endothelial ENaC has been associated with NO production and inhibition of eNOS, but vasodilation in resistance arteries is also mediated by endothelial‐dependent hyperpolarization (EDH; Garland & Dora, 2017). The use of specific inhibitors such as L‐NAME, TRAM34, and paxilline would be required to separate the contributions of these pathways. Finally, our dietary and hormone manipulations likely affect blood pressure in the animals which could change endothelial function across our four groups due to changes in fluid shear stress and mechanical stretch of the vessels. However, our study focused on whether Ach sensitivity was altered in the presence of amiloride. Our method of using one vessel split in two controls for these changes.

In summary, our results suggest a dynamic role of ENaC in the endothelium. Our results suggest that the effects of ENaC on endothelial‐dependent vasodilation evolve over time when mice are on a HSD. An initial ENaC‐dependent enhancement of vasodilation changes to an ENaC‐dependent blunting of vasodilation, which may have detrimental consequences. Further studies are required to understand mechanisms underlying this ENaC‐dependent change in endothelial function and what role ENaC in the smooth muscle may play in mediating these responses. Our studies raise the possibility that vascular ENaC has a role in vascular dysfunction in the setting of salt‐sensitivity.

## CONFLICT OF INTEREST

The authors have no conflict of interest to declare.
