# Correction to “Effects of amiloride on acetylcholine‐dependent arterial vasodilation evolve over time in mice on a high salt diet”

**DOI:** 10.14814/phy2.70820

**Published:** 2026-03-17

Mutchler, S. M., & Kleyman, T. R. (2022). Effects of amiloride on acetylcholine‐dependent arterial vasodilation evolve over time in mice on a high salt diet. *Physiological Reports*, 10, e15255. https://doi.org/10.14814/phy2.15255

The American Physiological Society and The Physiological Society (UK) are issuing a Society Note to include an Editor Disclosure Statement that was accidentally omitted from this published article in *Physiological Reports*. It should have been part of the Conflict of Interest statement.

Editor Disclosure Statement

Thomas R. Kleyman, a co‐author on this article, was the Editor‐in‐Chief of the journal at the time the article was published. The Societies verify that Thomas R. Kleyman was not involved in the handling of the manuscript and did not have access to information regarding the peer‐review process or final disposition of the article. An alternate editor oversaw the peer‐review and decision‐making processes.

